# Telephonic Communication

**Published:** 1879-02

**Authors:** 


### Telephonic Communication.

By aid of the Edison telephone and the facilities afforded by the American District Telegraph Co., we are soon to be in direct speaking communication with our publishers and many of our professional and business friends, as well as hospitals and other public institutions.

———:o:———

THE DISINFECTION of hair brought from unknown or foreign sources for use in upholstery, seems to be necessary as a safeguard against infection of those employed in handling or using it, with charbon or malignant pustule, some cases of which have lately occurred in Massachusetts. A series of cases was reported to the Massachusetts Medical Society in 1870, and details of the cases are well described in the published communications of the Society for that year. A prolonged reference of the hair to a moist heat of 140° Fahrenheit or over and the use of sulphuric acid, are said by the *Boston Med. Jour.* to have been found effectual in disinfection of charbon virus by Davaine in reports in the Compt. Rend. Acad. des Sciences, of Sept. 29th, and Oct. 13th, 1873.

